# Predictive Equation for Peak Heart Rate and First Ventilatory Threshold Heart Rate in Patients With Coronary Heart Disease

**DOI:** 10.1155/crp/4446755

**Published:** 2026-06-23

**Authors:** Xianghui Zheng, Peiyao Wang, Shiyu Wang, Haoning Cui, Hu Tan, Lida Guan, Shujiang Zhang, Hongyan Zhang, Fang Wang, Xinyu Hou, Qifeng Li, Tianhui Cao, Yang Zheng, Xiaojun Wu, Jian Wu, Bo Yu

**Affiliations:** ^1^ Department of Cardiology, The Second Affiliated Hospital of Harbin Medical University, Harbin, China, hrbmush.edu.cn; ^2^ Department of Cardiac Rehabilitation Center, The Second Affiliated Hospital of Harbin Medical University, Harbin, China, hrbmush.edu.cn; ^3^ Key Laboratory of Myocardial Ischemia, Ministry of Education, Harbin Medical University, Harbin, China, hrbmu.edu.cn; ^4^ Department of Cardiology, The Second Affiliated Hospital, Army Medical University (Third Military Medical University), Chongqing, China; ^5^ Department of Cardiology, Yichun Central Hospital, Yichun, China; ^6^ Department of Cardiology, Jiansanjiang Hospital of Beidahuang Group, Jiamusi, China; ^7^ Department of Cardiology, The Second Affiliated Hospital of Qiqihar Medical University, Qiqihaer, Heilongjiang, China; ^8^ Department of Cardiology, Jilin Province FAW General Hospital, Jilin, China; ^9^ State Key Laboratory of Frigid Zone Cardiovascular Diseases (SKLFZCD), Harbin Medical University, Harbin, China, hrbmu.edu.cn

**Keywords:** cardiac rehabilitation, cardiopulmonary exercise testing, coronary heart disease, heart rate

## Abstract

**Background:**

Peak heart rate (HR peak) and first ventilatory threshold heart rate (HR VT1) guide exercise prescription formulation, but existing formulas lack accuracy in coronary heart disease (CHD) patients due to unaccounted pathophysiological differences. This study aimed to construct prediction equation for HR peak and HR VT1 in CHD patients.

**Methods:**

This was a multicenter retrospective study that included 14,465 cases of cardiopulmonary exercise test (CPET) data from CHD patients in 20 hospitals in China. Seventy percent of the cohort was divided into a development group (*n* = 10,125), and the remaining 30% served as a validation group (*n* = 4340). Stepwise multiple backward regression established HR peak and HR VT1 equations, with accuracy compared to traditional formulas.

**Results:**

Age, weight, resting heart rate (HR rest), CHD diagnostic category, and β‐blockers were included in the equation. The mean absolute percentage error (MAPE) of China‐CPET‐HR peak is 9.04%, with an adjusted coefficient of determination (*R* ^2^) of 0.399. For the China‐CPET‐HR VT1 formula, the MAPE is 7.32% and the adjusted *R* ^2^ is 0.509. The %HR peaks of the FOX, TANAKA, KETEYIAN, and China‐CPET‐HR peak formulas are 82 ± 11%, 79 ± 11%, 105 ± 13%, and 100 ± 11%, respectively.

**Conclusion:**

Based on CPET data from CHD patients, we developed prediction equations for HR peak and HR VT1. The prediction accuracy of these equations is significantly higher than others, which helps to formulate accurate individualized exercise prescriptions and rehabilitation training guidance for CHD patients.

## 1. Introduction

Despite significant progress in the management of coronary heart disease (CHD) over the past few decades, CHD remains the leading cause of death worldwide [1, 2]. Exercise‐based cardiac rehabilitation (CR) is recognized as a key component of comprehensive CHD management and is a Class I, Level A recommendation in international guidelines [3]. The 2024 European Society of Cardiology guidelines recommend at least 150 min of moderate‐intensity exercise or 75 min of vigorous‐intensity exercise weekly for patients with CHD [3]. When determining exercise intensity, target heart rate (THR)–based assessment methods are commonly used simple indicators [4].

THR, a core parameter for exercise prescription development, directly affects exercise effectiveness and the risk of cardiovascular events [5], and its setting is mainly based on peak heart rate (HR peak) and first ventilatory threshold heart rate (HR VT1). HR peak is usually defined as the highest heart rate attained during the maximal symptomatic restrictive cardiopulmonary exercise test (CPET) [6], which serves as a critical indicator for measuring exercise intensity and is applicable in the development of exercise prescriptions [7]. The European Society of Cardiology defines moderate‐intensity exercise as exercise performed at 40%–69% of HR peak, whereas high‐intensity exercise is defined as exercise performed at 75%–90% of HR peak [7]. In 2022, the European Association of Preventive Cardiology (EAPC) issued a position statement identifying the ventilatory threshold (VT) derived from CPET as the gold standard for determining aerobic exercise intensity [8]. The intensity zone around the first ventilatory threshold (VT1) is generally regarded as the lower limit of moderate intensity and can serve as an initial marker for defining actual exercise intensity in patients with cardiovascular disease [9, 10]. Therefore, it is critical to assess HR peak and HR VT1 to tailor exercise programs for CHD populations.

Recent research has focused primarily on establishing prediction equations for HR peak, such as the FOX [11] and TANAKA [12] equations, all of which predict HR peak on the basis of age. However, HR peak is influenced by a variety of physiological functions with significant individual heterogeneity, and linear equations that rely solely on the variable of age are systematically biased [13]. Second, these formulas were developed on the basis of data from healthy populations and fail to fully account for the pathophysiological differences in patients with CHD, thus demonstrating limited efficacy in the CHD population. Moreover, the KETEYIAN formula [14] was developed on the basis of a small sample of Western patients with heart failure (HF), and direct application to patients with CHD may lead to deviations. Additionally, previous studies in the HR VT1 research field have predominantly estimated HR VT1 through percentages of HR peak predictive values, and a large‐sample evidence‐based basis for the direct prediction of HR VT1 is lacking.

Therefore, the aim of this study was to develop prediction formulas for HR peak and HR VT1 for the population with CHD and compare the accuracy of the new formulas with that of the existing formulas to provide an evidence‐based basis for the precise formulation of exercise prescriptions for patients with CHD.

## 2. Materials and Methods

### 2.1. Study Population

This was a multicenter retrospective study that included 14,465 cases of CPET data from CHD patients from CR centers in 20 hospitals in China between January 2018 and December 2024. Data from each participating CR center passed a rigorous review, and these hospitals were geographically distributed across seven regions in China: north, east, central, south, southwestern, northeastern, and northwestern China (nine in northeastern China, three in northwestern China, two in eastern China, two in southern China, two in northern China, one in central China, and one in southwestern China). The inclusion criteria were as follows: (1) patients with a confirmed diagnosis of CHD; (2) patients aged between 30 and 79 years; (3) patients who had undergone symptom‐limited CPET; (4) patients for whom the CPET was performed via cycle ergometry; and (5) patients with a peak respiratory exchange ratio (RER) ≥ 1.0. The exclusion criteria were as follows: (1) patients with concurrent atrial fibrillation and (2) patients whose CPET data showed interference. The specific definitions of the CHD diagnostic categories are as follows: the myocardial infarction (MI) category included patients with a reported definite diagnosis of MI, regardless of whether they had undergone percutaneous coronary intervention (PCI). The PCI category excluded patients with a reported history of MI, and the stable angina (SA) category excluded patients with a reported history of either MI or PCI.

In this study, all the data were anonymized historical data that did not involve patient intervention or additional biological sample collection. All personal information was de‐identified. The study was conducted in accordance with the Declaration of Helsinki and approved by the Second Affiliated Hospital of Harbin Medical University (protocol code KY2025‐031 in February 2025).

### 2.2. CPET

All CPET procedures performed in the rehabilitation centers of this study adhered to international standards for adult CPET [15, 16]. The participants completed a maximal, symptom‐limited CPET on a cycle ergometer with breath‐by‐breath gas analysis and electrocardiographic monitoring. The ramp protocol was chosen as the cycling protocol, and the incremental load per minute was the value of the expected maximum power divided by 8–12, with a target test time of 8–12 min until the patient reached maximal exertion or developed limiting symptoms that required the experiment to be terminated. The CPET termination criteria were as follows [17]: (1) moderate‐to‐severe angina pectoris; (2) symptoms of dizziness, ataxia, cyanosis or pallor, severe fatigue, or dyspnea; (3) electrocardiographic findings of horizontal or downsloping ST‐segment depression ≥ 0.2 mV in adjacent leads for ≥ 2 min, or coved ST‐segment elevation ≥ 0.1 mV; (4) severe arrhythmias, such as a second‐ to third‐degree atrioventricular block, ventricular tachycardia, frequent premature ventricular contractions, or new‐onset atrial fibrillation with rapid ventricular response; (5) a decrease in systolic blood pressure drop ≥ 10 mmHg (1 mmHg = 0.133 kPa) or persistent hypotension below baseline during workload increment, a systolic blood pressure ≥ 220 mmHg and/or diastolic blood pressure ≥ 110 mmHg; (6) a significant decline in cycling speed due to lower limb weakness, muscle pain, or spasm; and (7) participant request to terminate the exercise test. CPET data, including resting heart rate (HR rest), HR VT1, and HR peak, were determined according to standardized principles. The VT1 was identified via V‐slope analysis of VO_2_ and VCO_2_ [18], with the heart rate measured at the VT1 time point defined as HR VT1. HR values were consistently extracted using a 10‐s time‐averaged method rather than instantaneous single‐point readings. This approach was employed to minimize random variability and ensure full data comparability across all testing sites. Notably, all tests were re‐evaluated by experts blinded to patients’ clinical features, and at least one of the local CPET experts underwent a training program.

### 2.3. Classification of the Exercise‐Induced HR Response

This study included different prediction formulas for HR peak. HR peak data were also analyzed as a percentage of the maximum predicted values according to the following standard formulas:

(1) %HR peakFOX=HR peak220−age ×100,

(2) %HR peakTANAKA=HR peak208−0.7∗age ×100,

(3) %HR peakKETEYIAN=HR peak114+0.5∗HR rest−0.5∗Age×100.

### 2.4. Formula Development and Verification

Seventy percent of the participants were allocated to the formula development group, whereas 30% were reserved for the validation group. Stepwise multiple backward regression analysis was employed to identify predictors for the China‐CPET‐HR peak/HR VT1 equations. All independent variables in the final models demonstrated partial *R* ^2^ values ≥ 0.01.

### 2.5. Statistical Analysis

Data conforming to a normal distribution are expressed as mean ± standard deviation, and significant differences between two groups were compared using *t* test. Nonnormally distributed data are presented as medians or interquartile ranges, and the Mann‒Whitney *U* test was used to assess significant differences between groups. Categorical variables are expressed as frequencies (percentages), and the chi‐square test was applied to compare significant differences between groups. A two‐sided *p* < 0.05 was considered statistically significant. The mean absolute percentage error (MAPE) of each formula was calculated and compared, with the MAPE defined as (average absolute percent error for each period − actual value)/(actual value). All the statistical analyses were performed using R 4.3.2.

## 3. Results

A total of 14,465 cases of CPET data from CHD patients were included in this study, covering different ages, sexes, heights, weights, and other basic characteristics (Table 1). The median age was 56.89 years, and 30.9% of the participants were female. A total of 10,125 (70%) CPET data points were used for model development, and 4340 (30%) were used for validation. There were no significant differences between the two groups of participants in terms of age, height, weight, BMI, or sex (*p* > 0.05). With respect to the diagnostic classification of CHD, there was no significant difference between the two groups in terms of MI, PCI, and SA. The relevant indices of the CPET showed that the overall mean HR peak values was 133.38 ± 19.60 bpm, and the overall mean HR VT1 values was 107.70 ± 14.90 bpm. The differences in the key indicators of HR peak and HR VT1 between the two groups were not statistically significant.

**TABLE 1:** Main clinical variables of the study population.

Characteristics	Overall (*n* = 14,465)	Development (*n* = 10,125)	Validation (*n* = 4340)	*p* value
Age (year)	56.91 ± 10.04	58.00 (51.00–64.00)	58.00 (50.00–64.00)	0.482
Height (cm)	168.18 ± 7.72	170.00 (162.00–174.00)	170.00 (162.00–174.00)	0.706
Weight (kg)	72.10 ± 12.37	71.00 (63.00–80.00)	71.00 (63.15–80.00)	0.906
BMI (kg/m^2^)	25.39 ± 3.68	25.20 (23.20–27.50)	25.20 (23.10–27.40)	0.948
Sex				0.292
Male	9990 (69.1%)	7020 (69.3%)	2970 (68.4%)	
Female	4475 (30.9%)	3105 (30.7%)	1370 (31.6%)	
MI	3197 (22.1%)	2229 (22.0%)	968 (22.3%)	0.717
PCI	3999 (27.7%)	2798 (27.6%)	1201 (27.7%)	0.979
SA	6645 (45.9%)	4645 (45.9%)	2000 (46.1%)	0.834
Other	624 (4.3%)	453 (4.5%)	171 (3.9%)	0.160
Beta‐blockers	4899 (33.9%)	3443 (34.0%)	1456 (33.5%)	0.608
CPET				
RER peak	1.14 ± 0.09	1.12 (1.07–1.19)	1.12 (1.07–1.19)	0.817
HR peak, bpm	133.38 ± 19.60	132.00 (119.00–146.00)	133.00 (120.00–146.00)	0.357
HR VT1, bpm	107.70 ± 14.90	107.00 (97.00–117.00)	106.00 (97.00–117.00)	0.313
HR rest, bpm	83.51 ± 12.29	83.00 (75.00–92.00)	83.00 (75.00–92.00)	0.106
VO_2_ peak, mL/kg/min	18.36 ± 4.34	17.71 (15.20–20.86)	17.81 (15.20–20.83)	0.590
VO_2_ AT, mL/kg/min	11.95 ± 2.71	11.53 (10.10–13.41)	11.54 (10.10–13.40)	0.878
SBP rest, mmHg	130.70 ± 20.94	130.00 (116.00–145.00)	130.00 (116.00–145.00)	0.503
DBP rest, mmHg	79.79 ± 11.85	79.00 (71.00–88.00)	79.00 (72.00–88.00)	0.231

*Note:* HR VT1, heart rate at first ventilatory threshold; HR peak, heart rate at peak exercise; VO_2_ AT, oxygen uptake at anaerobic threshold; VO_2_ peak, peak oxygen uptake; MET, metabolic equivalent.

Abbreviations: BMI, body mass index; DBP rest, diastolic blood pressure at rest; HR rest, heart rate at rest; MI, myocardial infarction, PCI, percutaneous coronary intervention; RER, respiratory exchange ratio; SA, stable angina; SBP rest, systolic blood pressure at rest.

Stepwise multiple backward regression analyses revealed that age, weight, HR rest, type of CHD diagnosis, and β‐blockers had significant effects on the China‐CPET‐HR peak and China‐CPET‐HR VT1 (*p* < 0.001) (Table 2). China‐CPET‐HR peak = 113.62 − (0.58 ∗ age [years]) − (0.16 ∗ weight [kg]) + (0.69 ∗ HR rest [bpm]) + (3.91 ∗ MI [yes = 1; no = 0]) + (6.65 ∗ PCI [yes = 1; no = 0]) + (12.34 ∗ SA [yes = 1; no = 0]) − (3.53 ∗ β‐blockers [yes = 1; no = 0]), with accurate data (SEE = 15.06 bpm, adjusted *R* ^2^ = 0.399). China‐CPET‐HR VT1 = 64.18 − (0.28 ∗ age [years]) − (0.11 ∗ weight [kg]) + (0.76 ∗ HR rest [bpm]) + (1.99 ∗ MI [yes = 1; no = 0]) + (2.97 ∗ PCI [yes = 1; no = 0]) + (6.30 ∗ SA [yes = 1; no = 0]) − (0.72 ∗ β‐blockers [yes = 1; no = 0]) (SEE = 10.39 bpm, adjusted *R* ^2^ = 0.509).

**TABLE 2:** Main clinical variables independently associated at HR peak and HR VT1 in the development cohort sample.

Characteristics	RMSE	*R* ^2^	*p* value
HR peak			
HR rest	17.1	0.246	< 0.001
Age	16.2	0.323	< 0.001
SA	15.6	0.376	< 0.001
Weight	15.4	0.386	< 0.001
PCI	15.4	0.390	< 0.001
MI	15.4	0.391	< 0.001
Beta‐blockers	15.3	0.397	< 0.001
HR VT1			
HR rest	11.2	0.443	< 0.001
Age	10.9	0.473	< 0.001
SA	10.6	0.496	< 0.001
Weight	10.5	0.503	< 0.001
PCI	10.5	0.504	< 0.001
MI	10.5	0.505	< 0.001
Beta‐blockers	10.5	0.505	0.003

*Note:* See Table 1 for abbreviations. Note that only variables with an *R* ^2^ ≥ 0.01 are reported.

Table 3 shows the comparison between the China‐CPET‐HR peak and traditional formulas. The China‐CPET‐HR peak formula exhibited an *R* ^2^ of 0.399, an SEE of 15.06 bpm, and a MAPE of 9.04%. The traditional HR peak prediction formulas, such as the FOX, TANAKA, and KETEYIAN formulas, had *R* ^2^ values of 0.115, 0.115, and 0.316, with MAPE values of 24.94%, 28.66% and 9.79%, respectively. Compared with the traditional FOX, TANAKA, and KETEYIAN formulas, the China‐CPET‐HR peak formula yielded lower MAPE values.

**TABLE 3:** Historical and new equations for estimating HR peak and new equations for estimating HR VT1 and related accuracy data.

HR peak, historical equations	Equations	*R* ^2^	SEE, beats·min^−1^	MAPE (%)
HR peak_FOX_	220‐age	0.115	34.94	24.94
HR peak_TANAKA_	208–0.7 ∗ age	0.115	39.17	28.66
HR peak_KETEYIAN_	114 + (0.5 ∗ HR rest) − (0.5 ∗ age)	0.316	17.36	9.79

*HR peak and HR VT1 New equation*				
China‐CPET‐HR peak	HR peak = 113.62 − (0.58 ∗ age (years)) − (0.16 ∗ weight (kg)) + (0.69 ∗ HR rest (bpm)) + (3.91 ∗ MI [yes = 1; no = 0]) + (6.65 ∗ PCI [yes = 1; no = 0]) + (12.34 ∗ SA [yes = 1; no = 0]) − (3.53 ∗ β‐blockers [yes = 1; no = 0])	0.399	15.06	9.04
China‐CPET‐HR VT1	HR VT1 = 64.18 − (0.28 ∗ age (years)) − (0.11 ∗ weight (kg)) + (0.76 ∗ HR rest (bpm)) + (1.99 ∗ MI [yes = 1; no = 0]) + (2.97 ∗ PCI [yes = 1; no = 0]) + (6.30 ∗ SA [yes = 1; no = 0]) − (0.72 ∗ β‐blockers [yes = 1; no = 0])	0.509	10.39	7.32

*Note:* SEE, standard error of the estimate; For other abbreviations, see Table 1.

Abbreviation: MAPE = mean absolute percentage error.

Table 4 shows all the HR data expressed either as absolute values or as percentages of the maximum value predicted according to each of the different equations analyzed in the actual study. For HR peak, the %HR peak values for the FOX, TANAKA, and KETEYIAN equations were 82 ± 11%, 79 ± 11%, and 105 ± 13%, respectively, whereas the %HR peak for the China‐CPET equation was 100 ± 11% and %HR VT1 was 100 ± 10%.

**TABLE 4:** Percentage of actual to predicted values for HR peak and HR VT1.

Variables	Equations
HR rest (bpm)	84 ± 12
HR peak (bpm)	134 ± 19
HR peak (FOX) (%)	82 ± 11
HR peak (TANAKA) (%)	79 ± 11
HR peak (KETEYIAN) (%)	105 ± 13
HR peak (China‐CPET‐MHR) (%)	100 ± 11
HR VT1 (bpm)	108 ± 15
HR VT1 (China‐CPET‐HR VT1) (%)	100 ± 10
HR rest (bpm)	84 ± 12

*Note:* %HR VT1 = [actual HR VT1/predicted HR VT1] × 100. For abbreviations, see Table 1.

**FIGURE 1:**
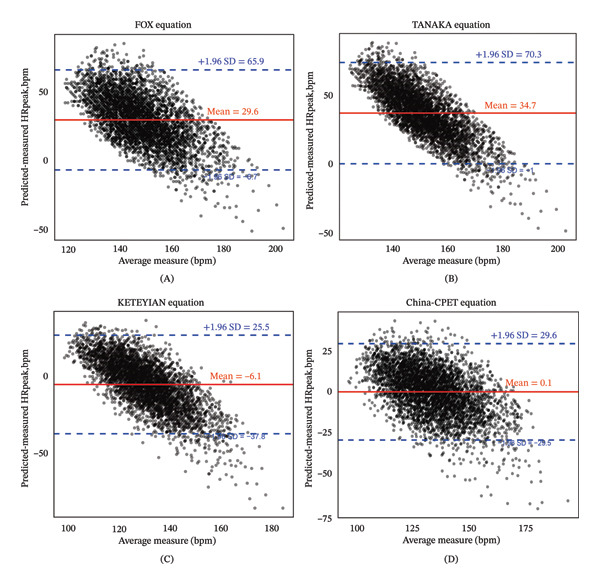
The consistency between the predicted maximum heart rate values and the actual values of the four equations, namely, FOX (A), TANAKA (B), KETEYIAN (C), and China‐CPET (D), was compared in the validation set. The horizontal axis represents the average value of the predicted value and the actual measured value, and the vertical axis represents the difference between the predicted value and the actual measured value. The red solid line (Mean) indicates the mean bias. If mean > 0, it means that the heart rate predicted by the equation is higher than the actual heart rate; if Mean < 0, it means that the heart rate predicted by the equation is lower than the actual heart rate. The blue dashed lines (± 1.96 SD) are the limits of agreement, reflecting the dispersion range of approximately 95% of the data.

The comparison results of the consistency between the predicted maximum heart rate values and the actual values of the four equations show that, as presented in Figure 1 and Figure S1, the mean value of the China‐CPET equation is closer to 0 in both the validation set and the training set. Additionally, its data points are most concentrated within the limits of agreement, indicating that it has the smallest deviation between the predicted heart rate and the actual heart rate, as well as the best consistency.

## 4. Discussion

HR peak and HR VT1 play key roles in exercise physiology, but their direct measurement is limited by the lack of equipment and technical personnel and the effect of a patient’s own physical state; thus, building predictive formulas is a better solution. Existing prediction formulas for HR peak widely used in clinical practice have been developed and validated in Western populations, introducing systemic physiological parameter biases when applied to Asian populations. Park et al. conducted a validation study in a healthy Korean population (aged 7–55 years) and demonstrated substantial discrepancies between the predicted and measured values using conventional formulas [19]. Notably, most exercise prescriptions rely on HR AT, yet large‐scale HR VT1 prediction formulas remain unavailable. Small‐sample studies based on the Chinese population revealed that the traditional prediction formula for HR peak (220‐age/208‐0.7 ∗ age) was not applicable to the Chinese population, and the %HR peak was only 75.9% and 73.4% [20], respectively. Therefore, we constructed a prediction equation for HR peak and HR VT1 that is suitable for the CHD population on the basis of a cohort of Chinese patients with CHD.

Previous studies have demonstrated that age [21], body weight [22], and HR rest [14] significantly influence HR peak, with these parameters being incorporated into established predictive formulas [12, 23]. Existing research [24] highlights anthropometric differences across ethnic groups in terms of baseline characteristics such as height and weight. Furthermore, a U.S. population–based survey revealed statistically significant interethnic disparities in HR rest [25]. Different ethnic groups exhibit variations in heart rate regulatory genes due to their unique genetic backgrounds [26]. Therefore, the above‐mentioned indicators were prioritized in constructing the predictive formulas for HR peak and HR VT1 in this study. Consistent with previous work, the results of this study also revealed that age, body weight, and HR rest were associated with the predictive formulas (*p* < 0.001). After these indicators were incorporated into the predictive formulas for HR peak and HR VT1, the *R* ^2^ values significantly increased.

Considering the pathophysiological characteristics of CHD, we added CHD diagnostic types including MI, PCI, and SA, to the predictive formula on the basis of traditional factors. Previous studies have shown differences in HR between patients with CHD and healthy individuals [27]. HR changes in patients with CHD are associated primarily with atherosclerosis, myocardial ischemia, autonomic nervous system dysfunction, and cardiac structural and functional changes [28]. As the most widely used predictive models in clinical practice, the FOX and TANAKA formulas were derived from healthy populations, and fail to account for the interference of CHD status with HR [29]. They cannot fully capture the subtle age‐related changes in HR peak among patients with CHD. In this study, the %HR peak values of the FOX and TANAKA formulas were 82 ± 11% and 79 ± 11%, respectively, which significantly deviated from the measured values. If the corresponding HR peak prediction results are directly applied to patients with CHD, it may lead to reduced exercise safety and an increased risk of cardiovascular events. After MI, PCI, and SA were incorporated into the formula, the RMSE and *R* ^2^ significantly improved, reaching 15.4 and 0.391, respectively. The KETEYIAN equation demonstrated better performance than the FOX and TANAKA formulas, with an *R* ^2^ of 0.316, a MAPE of 9.79%, and a %HR peak of 105 ± 13%. However, the KETEYIAN equation is primarily used in patients with HR, a complex cardiac syndrome involving multiple mechanisms such as cardiac systolic and diastolic dysfunction, neuroendocrine activation, and myocardial remodeling, which affects HR differently from CHD [30]. Additionally, variable selection for KETEYIAN equation is still limited to age and HR rest, without covering the effect of CHD history. The HR peak equation developed in this study retains basic physiological indicators such as age and body weight while further incorporating cardiovascular disease‐specific variables such as CHD diagnostic types, making it more suitable for patients with CHD. The %HR peak of this formula reaches 100 ± 11%, which is closer to the measured values than those of the above three formulas.

Beta‐blockers act as negative chronotropic agents: by inhibiting sympathetic nerve activity, they lower both resting and maximal heart rates while improving prognosis in patients with CHD. Guideline recommendations support their use [31]. Several earlier studies have confirmed that beta‐blocker use, type, and dosage each independently influence predicted heart rate values [20, 29]. Importantly, adjusting for HR rest alone does not fully account for the effect of beta‐blockers, which introduces bias and reduces accuracy when conventional heart rate prediction equations are applied clinically. In the present study, beta‐blocker use emerged as an independent determinant of both HR peak and heart rate at the VT1. Adding beta‐blocker status to the prediction model raised the *R* ^2^ value of the China‐CPET‐HR peak equation to 0.397, reflecting better explanatory power and predictive precision. With this adjustment, the model aligns more closely with the physiological characteristics of CHD patients receiving standard medical therapy, thereby improving its clinical applicability. Nevertheless, future studies can further expand the sample size and more carefully analyze the specific impact of different types of beta‐blockers on the research results to enhance the persuasiveness of the conclusions.

An additional clinical question addressed in this study pertains to advancing CR insights for patients with CHD. Exercise training constitutes the cornerstone of cardiovascular rehabilitation programs, with critical objectives extending beyond event reduction to encompass symptom amelioration and psychosocial well‐being [1]. A pivotal challenge lies in delineating the true exercise intensity domain, where the AT—marking the aerobic‒anaerobic metabolic transition—serves as the physiological benchmark. Current guidelines predominantly prescribe an exercise intensity that targets HR VT1 [10]. However, previous methodologies have been constrained by deriving HR VT1 indirectly from predicted HR peak values [32], and validated tools for direct HR VT1 estimation are lacking. To address this gap, we developed a novel HR VT1 prediction equation specifically for patients with CHD that demonstrated robust performance metrics: an adjusted *R* ^2^ of 0.509, a MAPE of 7.32%, and a %HR VT1 of 100 ± 10%. In this study, we also found that when the same relevant factors were used, the MAPE of the HR peak prediction formula was 9.04%, which was less accurate for predicting HR VT1. This may be because we used a symptom‐limited CPET, in which some patients experienced symptoms such as myocardial ischemia during exercise and terminated the test early. However, most patients could reach the VT1, which is less affected by subjective factors. Therefore, our formula is more accurate for predicting HR VT1.

We demonstrated that age‐predicted equations performed worse in participants with risk factors for CHD than in those without for CHD (Table 3), which is a clinically meaningful observation. Owing to concerns about the increased risk of complications during maximal exercise testing [33], healthcare providers and exercise specialists often opt for submaximal exercise testing protocols in this population. Routine use of fixed percentages of the predicted HR peak (e.g., 85% or 100% of age‐predicted HR peak formulas) as submaximal test endpoints in older adults may result in THRs that represent maximal intensity for some individuals, are unattainable for others, or only reflect submaximal levels for some [34]. Therefore, the use of HR peak prediction formulas may result in inaccurate assessments of exercise capacity, and such errors could have significant clinical implications across different clinical scenarios. Growing evidence indicates that aerobic fitness is not only a strong predictor of health outcomes [35] but also an effective tool for evaluating the prognosis of CHD. Therefore, while HR peak prediction formulas offer convenience in assessing exercise intensity, it is essential to ensure that participants reach their subjective fatigue limit, and preset heart rates should be avoided as criteria for terminating the test [36].

Exercise‐based rehabilitation improves clinical outcomes in patients with CHD, where precision in heart rate prediction enables the design of individualized exercise prescriptions. For moderate‐intensity exercise, the predicted HR VT1 can be used to ensure that patients maintain a heart rate below this level during exercise, thereby avoiding excessive fatigue and lactic acid accumulation and improving their exercise safety [37]. For high‐intensity exercise, accurate HR peak prediction helps set reasonable high‐intensity interval targets, which can improve patient compliance and improve cardiopulmonary function more effectively [38]. Generally, patients in the early stage of MI or with complex blood vessels after PCI are more suitable for exercise prescription formulation on the basis of HR VT1. In recent years, high‐intensity interval training (HIIT) programs have been regarded as alternative exercise modalities for low‐risk patients [39]. Current studies have shown that patients with CHD with stable symptoms benefit more from HIIT [40], so the combination of HR peak and HR VT1 can be considered in the formulation of exercise prescriptions for these patients. Accurate heart rate monitoring and control can reduce these risks, and prediction equations help set safe heart rate upper limits to prevent cardiovascular events caused by excessive exercise [41]. In future clinical trials of remote or home‐based CR, the current formulas may be referenced for exercise prescriptions for patients with CHD, which not only prevents insufficient exercise intensity from failing to achieve rehabilitation effects but also avoids excessive exercise intensity from increasing the risk of cardiovascular events, ultimately guiding optimal exercise training in a more effective manner. When CPET is unavailable, field‐based tests can still offer supplementary clinical information to inform the use of the proposed equations. Consider the 6‐min walk test (6MWT): it is straightforward, cheap, and commonly employed in CR. Among patients with stable coronary artery disease receiving beta‐blockers, the heart rate recorded at the end of the 6MWT agrees acceptably with the heart rate at VT1, which suggests that the test may help guide the prescription and monitoring of aerobic exercise intensity [42, 43]. Another practical alternative is the talk test–free, equipment‐free, and workable method for regulating exercise intensity. Evidence indicates that the talk test correlates with the ventilatory threshold and is highly valuable in CR, notably in resource‐limited or home‐based settings [44, 45]. These tools should be seen as complementary to, not a replacement for, threshold assessment derived from CPET. However, despite the convenience of predictive equations, individual variations exist among patients with CHD, and some patients may experience myocardial ischemia during exercise. Therefore, when formulating exercise prescriptions, we still recommend prioritizing the CPET for assessing exercise intensity.

### 4.1. Limitation

CPET data from 14,465 patients with CHD across 20 Chinese hospitals were utilized in this study, establishing the largest derivation cohort to date for nonexercise prediction equations for HR peak and HR VT1 in CHD populations. However, several limitations warrant consideration. First, although CHD diagnostic types were incorporated into the prediction equations, the improvement in *R* ^2^ values remained relatively limited. Future research should include more comprehensive clinical parameters to further increase the predictive accuracy of the formulas. Second, detailed data on habitual physical activity levels were not systematically collected, and subgroup analysis was not performed to determine whether the equation was applicable to people with exercise habits. Additionally, the temporal interval between cardiovascular event occurrence and CPET assessment remains uncharacterized. Finally, as a retrospective study, some unmeasured confounding factors may exist, and prospective cohort studies are still needed to validate the findings.

## 5. Conclusion

Through a large‐scale, multicenter retrospective analysis of patients with CHD, this study established validated predictive equations for HR peak and HR VT1 by incorporating age, body weight, HR rest, CHD diagnostic subtypes, and β‐blockers. These population‐specific formulas provide evidence‐based HR targets for optimizing exercise intensity prescription during cardiac rehabilitation.

**Nomenclature**
VT1: First ventilatory threshold
CHD: Coronary heart disease
CPET: Cardiopulmonary exercise test
CR: Cardiac rehabilitation
HR VT1: First ventilatory threshold heart rate
HR rest: Resting heart rate
HR peak: Peak heart rate
THR: Target heart rate

## Funding

This study was supported by funding from the National Key R&D Program of China (Grant No. 2016YFC1301105).

## Conflicts of Interest

The authors declare no conflicts of interest.

## Supporting Information

Additional supporting information can be found online in the Supporting Information section.

## Supporting information


**Supporting Information** Figure S1 The consistency between the predicted maximum heart rate values and the actual values of the four equations, namely, FOX (A), TANAKA (B), KETEYIAN (C), and China ‐ CPET (D), was compared in the training set. The horizontal axis represents the average value of the predicted value and the actual measured value, and the vertical axis represents the difference between the predicted value and the actual measured value. The red solid line (Mean) indicates the mean bias. If Mean > 0, it means that the heart rate predicted by the equation is higher than the actual heart rate; if Mean < 0, it means that the heart rate predicted by the equation is lower than the actual heart rate. The blue dashed lines (±1.96 SD) are the limits of agreement, reflecting the dispersion range of approximately 95% of the data.

## Data Availability

The data that support the findings of this study are available from the corresponding author upon reasonable request.
