# Supplementary figures and images for: Predictive Equation for Peak Heart Rate and First Ventilatory Threshold Heart Rate in Patients With Coronary Heart Disease
Source: Cardiol Res Pract. 2026 Jun 23;2026:4446755. doi: 10.1155/crp/4446755 (PMC13288608; doi:10.1155/crp/4446755)

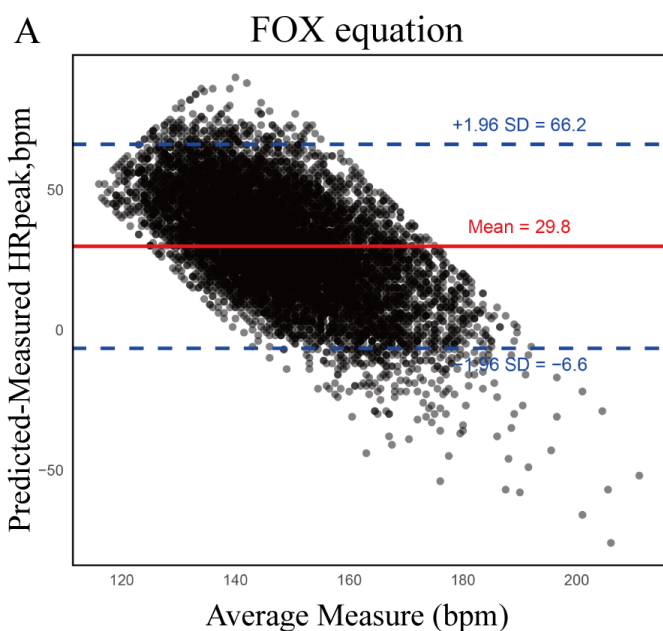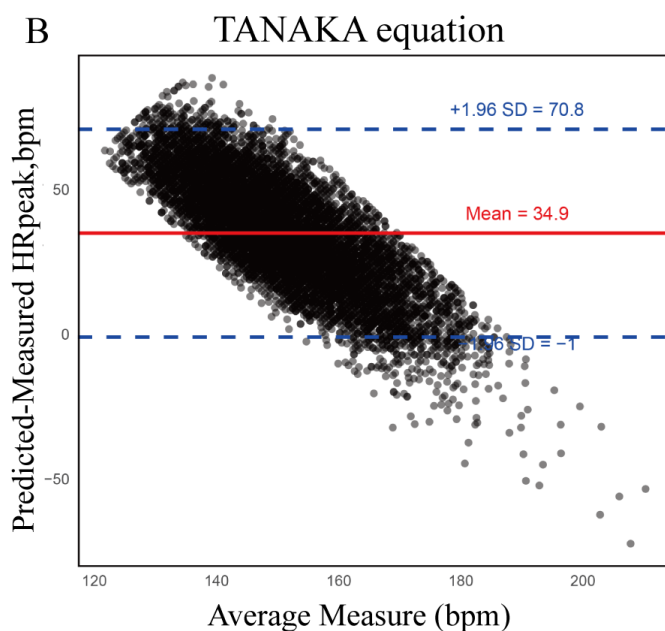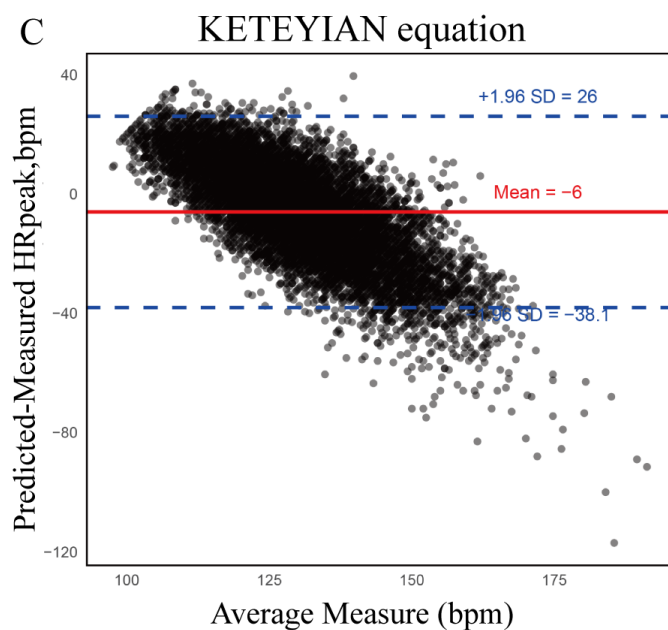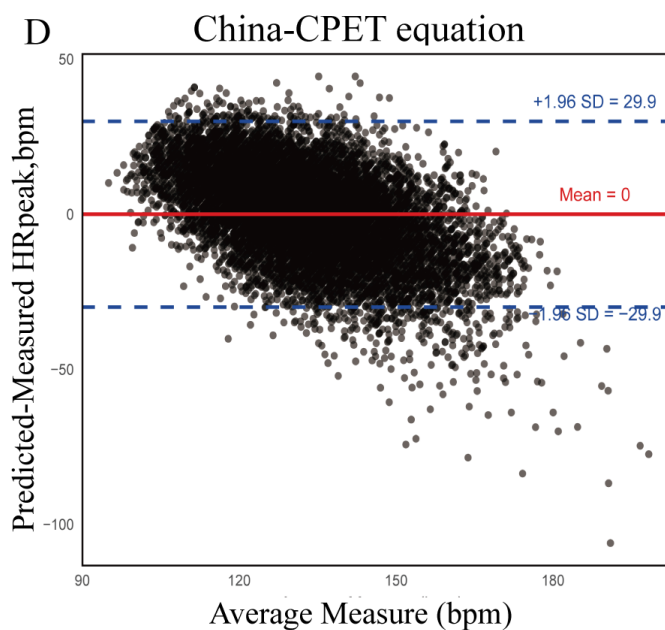

Supplement: Supplementary file 1 — Supporting Information Figure S1 The consistency between the predicted maximum heart rate values and the actual values of the four equations, namely, FOX (A), TANAKA (B), KETEYIAN (C), and China ‐ CPET (D), was compared in the training set. The horizontal axis represents the average value of the predicted value and the actual measured value, and the vertical axis represents the difference between the predicted value and the actual measured value. The red solid line (Mean) indicates the mean bias. If Mean > 0, it means that the heart rate predicted by the equation is higher than the actual heart rate; if Mean < 0, it means that the heart rate predicted by the equation is lower than the actual heart rate. The blue dashed lines (±1.96 SD) are the limits of agreement, reflecting the dispersion range of approximately 95% of the data. [file CRP-2026-4446755-s001.pdf]
